# Editorial: Pathogen suppression by plant-associated microbiota

**DOI:** 10.3389/fpls.2025.1604449

**Published:** 2025-04-14

**Authors:** Ariel Herrera-Vásquez, Rudolf Schlechter, Grace Armijo-Godoy, Mariela Ines Monteoliva

**Affiliations:** ^1^ Centro de Biotecnología Vegetal, Facultad de Ciencias de la Vida, Universidad Andres Bello, Santiago, Chile; ^2^ Department of Biology, Chemistry, Pharmacy, Institute of Microbiology and Dahlem Centre of Plant Sciences, Freie Universität Berlin, Berlin, Germany; ^3^ Agriaquaculture Nutritional Genomic Center (CGNA), Temuco, Chile; ^4^ Instituto de Fisiología de Recursos Genéticos Vegetales – Unidad de Estudios Agropecuarios (IFRGV-UDEA), Instituto Nacional de Tecnologia Agropecuaria - Consejo Nacional de Investigaciones Cientificas y Tecnicas (INTA-CONICET), Córdoba, Argentina

**Keywords:** plant microbiome, pathogen suppression, biocontrol agents, induced systemic resistance (ISR), microbiome dynamics, sustainable disease management, plant-microbe interactions

Plants interact with a broad spectrum of microorganisms, including non-pathogenic microbes, which play a crucial role in plant health, productivity, and stress resilience. While pathogens have been widely studied due to their detrimental impact on crops, research on beneficial microorganisms is increasing exponentially. Among them, the most extensively explored symbiotic interactions include nitrogen-fixing bacteria, mycorrhizal associations, and microbiota-driven pathogen suppression (Tharanath et al., 2024).

Microbial pathogen suppression operates through two primary mechanisms. The first involves direct antagonism, where beneficial microbes parasitize or compete with pathogens for resources, produce antimicrobials, or disrupt quorum sensing. The second mechanism involves the indirect suppression of pathogens via priming or Induced Systemic Resistance (ISR) in the host plant. During ISR, non-pathogenic microbes trigger plant immune responses, modulating hormonal signaling and enhancing pathogen resistance. These mechanisms are highly interconnected and influenced by external conditions such as soil composition, microbial activity, and community structure. Microbial consortia may interact synergistically to suppress plant diseases through collective effects within the plant-microbiome-environment system (Maciag et al., 2023).

Microbiome manipulation to suppress pathogens is an under-explored strategy to improve crop health and yield. There is an urgent need for sustainable disease management solutions, intensified by pathogen-resistance acceleration and even more by climate change. This Research Topic compiles studies examining the ecological, physiological, molecular, and functional aspects of pathogen suppression by plant microbiota, with a focus on symbiotic interactions.

**Figure 1:**
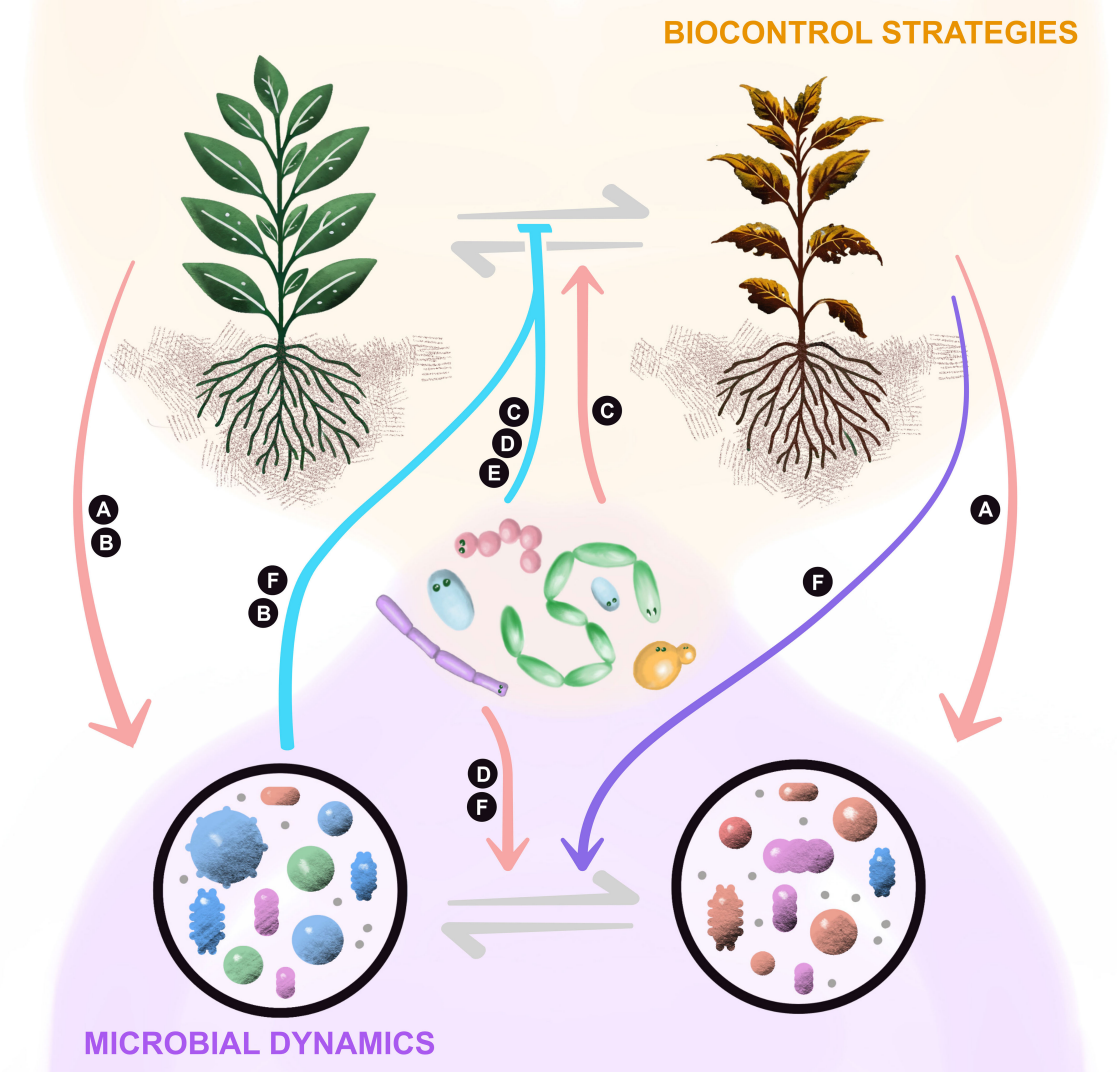
Key findings from studies on pathogen suppression by plant-associated microbiota. The articles in this Research Topic present the pathogen-suppression activity of plant-associated microorganisms through microbe-microbe interactions (pairwise or community-level) or plant-microbe interactions via the plant systemic resistance. Biocontrol strategies use beneficial microbes to manage plant diseases, either through direct antagonism or enhancement of plant defenses. Complementarily, microbial dynamics studies reveal shifts in microbial communities associated with plant health status and disease resistance, along with modulation of phytohormone signaling in the host. **(A)** Tran et al. demonstrated the influence of plant genotype in the recruitment of soil microbiota in soybean, highlighting the role of the host driving microbial assembly. **(B)** Oeum et al. examined microbial communities in rice and described how healthy plants possess signature microbial communities that may suppress *Xanthomonas oryzae* infection. **(C)** Teng et al. explored biocontrol strategies and microbial dynamics triggered by *Pichia* sp. and *Klebsiella oxytoca*, which suppress *Phytophthora nicotianae* and *Ralstonia solanacearum* infections in tobacco. **(D)** De Troyer et al. evaluated the biocontrol potential of *Streptomyces rimosus* in mitigating disease symptoms caused by *Fusarium graminearum* in wheat, emphasizing its role in stabilizing the wheat ear microbiome. **(E)** Elmeihy et al. described the biocontrol activity of *Trichoderma viride* and *Azospirillum brasilense* against *Harpophora maydis* infections in maize, demonstrating their antagonistic activity through metabolite production and their influence on microbial dynamics, as well as plant physiological and agronomic traits. **(F)** Jung et al. reviewed the role of top-down and bottom-up mechanisms of induced systemic resistance (ISR) by beneficial microbes.

The six articles provide valuable methodological approaches and insights into how the plant microbiota contributes to pathogen suppression (**Figure 1**). Comparative microbiome analyses reveal how microbial diversity and composition influence pathogen suppression, while biocontrol studies that focus on one species demonstrate the efficacy of microbial inoculants in suppressing phytopathogens, enhancing plant defenses, and influencing the host microbiome. Additionally, an in-depth review highlights the role of ISR, linking microbiome dynamics to plant immune responses.

Tran et al. contribute to this Research Topic by analyzing the microbiomes of soybean roots, rhizosphere, and bulk soil, as well as community shifts in response to cyst nematode (*Heterodera glycines*) infections. Using 16S rRNA gene amplicon sequencing, this study illustrated how resistant and susceptible host genotypes differentially recruited microbes during nematode infection. *Flavisolibacter* and *Sphingomonas* genera were enriched in the rhizosphere, while *Dyella* was enriched in the roots of the resistant genotype. These findings shed light on beneficial microbial communities that may contribute to nematode suppression in crops.

Oeum et al. compared microbial communities of healthy rice leaves and those affected by bacterial leaf blight (*Xanthomonas oryzae* pv. *oryzae*). They identified *Methylobacterium* species as indicators of healthy leaves and demonstrated their biocontrol potential to reduce disease severity. These findings provide a framework for leveraging microbiome signatures as an effective disease monitoring and suppression strategy.

From microbiome studies, particular species could be isolated and identified to be used as biocontrol agents. Biocontrol microbes offer a promising and eco-friendly alternative to traditional chemical treatments, which can harm beneficial microbes and contribute to pathogen resistance and soil degradation.

Elmeihy et al. explored the combined biocontrol effect of *Trichoderma viride* and *Azospirillum brasilense* against *Harpophora maydis*, the causal agent of late wilt disease in maize. Their study highlights the antagonistic activity of these microbes through metabolite production and the improvement of physiological and agronomical traits.

Teng et al. investigated the role of the yeast *Pichia* sp. and the bacterium *Klebsiella oxytoca* in promoting tobacco growth, as well as suppressing bacterial wilt (*Ralstonia solanacearum*) and black shank (*Phytophthora nicotianae*) diseases. Their findings proved that composite microbial fertilizers can modulate soil microbial communities and enhance plant health.

De Troyer et al. assessed the effectiveness of *Streptomyces rimosus* in mitigating Fusarium head blight caused by *Fusarium graminearum* in wheat ears. Their study demonstrated the ability of *S. rimosus* to reduce disease severity and stabilize the wheat ear microbiome, shifting its composition closer to a healthy microbiome.

Lastly, Jung et al. reviewed the systemic plant defense signaling induced by beneficial microbes as an alternative approach for pathogen suppression instead of direct microbe- microbe interactions. The authors describe how rhizospheric microbes can stimulate plant defenses while environmental stressors (biotic and abiotic agents) trigger changes in the root exudates that recruit and reshape the microbiome, further reinforcing plant defenses. They highlight the role of phytohormones and other signaling molecules in coordinating microbial recruitment and plant immune responses. Those interconnected effects underscore the dynamic relationship between plant and microbial communities, positioning ISR as a key process in microbiome-driven plant protection strategies.

This Research Topic presented three complementary methodological approaches to study beneficial microbes. Altogether, they might allow the design of tailored bioinputs as effective alternatives to chemical treatments for pathogen suppression. In the context of climate change and pathogen-resistance evolution (both exacerbated by anthropogenic activity), the need for sustainable, microbiome-driven disease management solutions has never been more urgent.

Future research needs to deepen our knowledge to optimize microbiome-based disease management by integrating microbial consortia, synthetic biology, and precision agriculture technologies to take advantage of the microbes’ full potential. Today, the most revolutionary challenge is to learn how to fine-tune natural or synthetic microbial communities (comprising hundreds or even thousands of species) to improve crop health and performance despite the significant technical limitations (Toju et al., 2020). The Research Topic raises some questions and challenges that will contribute to fully harnessing the potential of microbiome-based disease management:

- How do plant developmental stages and physiological status shape the recruitment and function of beneficial microbes across the plant body?
- Is it viable and efficient to design synthetic communities combining multiple beneficial functions, or is it better to choose functionally specialized microbes with different pathogen-suppression mechanisms?
- How resilient and stable are synthetic or native microbial consortia introduced into complex field environments?
- How can we better characterize and manipulate the shoot-root axis and the bidirectional feedback between them in plant-microbe-pathogen interactions?
- What signals modulate shoot-root microbiome diversity, composition, and function?
- How can we balance ecological complexity and formulation simplicity in microbial-based products to ensure both efficacy and practicality for farmers?

Addressing these issues will refine current strategies and define the future of sustainable crop protection. In the context of climate change, leveraging plant-microbe interactions for disease suppression is a strategic and promising alternative for securing global food security and promoting sustainable agriculture.
